# Epstein-Barr Virus and Systemic Lupus Erythematosus

**DOI:** 10.1155/2012/370516

**Published:** 2012-07-03

**Authors:** Anette Holck Draborg, Karen Duus, Gunnar Houen

**Affiliations:** Department of Clinical Biochemistry and Immunology, Statens Serum Institut, Ørestads Boulevard 5, 2300 Copenhagen, Denmark

## Abstract

The etiology of SLE is not fully established. SLE is a disease with periods of waning disease activity and intermittent flares. This fits well in theory to a latent virus infection, which occasionally switches to lytic cycle, and EBV infection has for long been suspected to be involved. This paper reviews EBV immunobiology and how this is related to SLE pathogenesis by illustrating uncontrolled reactivation of EBV as a disease mechanism for SLE. Studies on EBV in SLE patients show enlarged viral load, abnormal expression of viral lytic genes, impaired EBV-specific T-cell response, and increased levels of EBV-directed antibodies. These results suggest a role for reactivation of EBV infection in SLE. The increased level of EBV antibodies especially comprises an elevated titre of IgA antibodies, and the total number of EBV-reacting antibody isotypes is also enlarged. As EBV is known to be controlled by cell-mediated immunity, the reduced EBV-specific T-cell response in SLE patients may result in defective control of EBV causing frequent reactivation and expression of lytic cycle antigens. This gives rise to enhanced apoptosis and amplified cellular waste load resulting in activation of an immune response and development of EBV-directed antibodies and autoantibodies to cellular antigens.

## 1. Systemic Lupus Erythematosus

Systemic lupus erythematosus (SLE) is a rare autoimmune disease with an incidence of 6–35 new cases per 100.000 per year and typically presents in women (90% of cases) in the reproductive age [1–3]. The American College of Rheumatology (ACR) updated the clinical criteria for the classification of SLE in 1997, stating that 4 out of 11 criteria should be present consecutively or simultaneously during a period of observation in order to classify SLE (Table 1) [4]. The criteria involve dermatologic symptoms including a butterfly rash on the malar region of the face, discoid rash, photosensitivity, and oral or nasopharyngeal ulcers. Additional criteria comprise arthritis, serositis, renal disorders, and neurologic disorders (including seizures or psychosis). Different hematologic disorders are also included: anemia, leucopenia, lymphocytopenia, and thrombocytopenia. The last two criteria are immunologic disorders including: the presence of antinuclear antibodies (ANAs), which are observed in 80–90% of SLE patients. Most common are autoantibodies directed against double-stranded DNA (dsDNA) (58–70% of SLE patients [2, 5]), but also antibodies to other nuclear components such as histones, Ro52, Ro60, La, and Sm are frequently found [3–6]. The clinical presentation of SLE is influenced by a variety of factors including ethnicity, gender, age, socioeconomic factors, and age of onset [1]. The typical course of the disease is illustrated by periods of disease flares alternating with waning disease activity, and the typical treatment of SLE consists of immunosuppressive medication, which clinically improves the condition of the patients [7].

The etiology of SLE is believed to be multifactorial with genetic and environmental factors, both contributing to the development of this very complex disease. SLE is concordant in 24% of monozygotic twins and approximately 2% of dizygotic twins [8], indicating a genetic contribution. Certain major histocompatibility complex (MHC) II alleles, including HLA-DR2 and HLA-DR3, have been indicated to serve as risk factors in the development of SLE [5], and various HLA-DQ and HLA-DR alleles have been shown to be associated with the production of specific autoantibodies and other clinical manifestations of SLE [5]. Numerous other genes have been shown to be associated with the SLE pathogenesis especially components of interferon pathways (e.g., *IRF5*, *STAT4,* and *SPP1*), which probably reflects general intrinsic immune deficiencies in SLE patients [9, 10].

Impaired T-cell proliferation, and abnormal cytokine production has also been demonstrated to play a role in SLE pathogenesis [11]. A T helper 1/T helper 2 cytokine imbalance is observed in SLE patients. An enhanced T helper 17 cell response has also been detected and correlated with disease activity in SLE patients, which suggests a role for interleukin-17 (IL-17) in the pathogenesis of SLE [12, 13].

Another risk factor for developing SLE is deficiencies in the classical complement pathway, especially C1q (93%) and C4 (75%) deficiency. C1q deficiency may be inherited or acquired as a result of the production of C1q autoantibodies, which can be detected in some SLE patients. This results in decreased clearance of apoptotic materials leading to accumulation of apoptotic blebs [14–17]. Nuclear autoantigens are clustered at the surface of these blebs. As they are recognized by the immune system as “nonself,” they may initiate autoimmune responses [18]. This gives rise to the production of autoantibodies directed against conserved cellular components. The production of autoantibodies results in the formation of circulating immune complexes. When the concentration and size of these complexes reach a critical level, they may deposit in the subendothelium inciting inflammation and tissue damage [16, 19].

Environmental risk factors for SLE development are ultraviolet radiation and certain drugs and chemicals [21–23], and infections are known to be major environmental factors. Especially Epstein-Barr virus (EBV) infection has been shown to be highly associated with the development of SLE, as presented in the following sections.

## 2. Epstein-Barr Virus Infection

**Figure 1:**
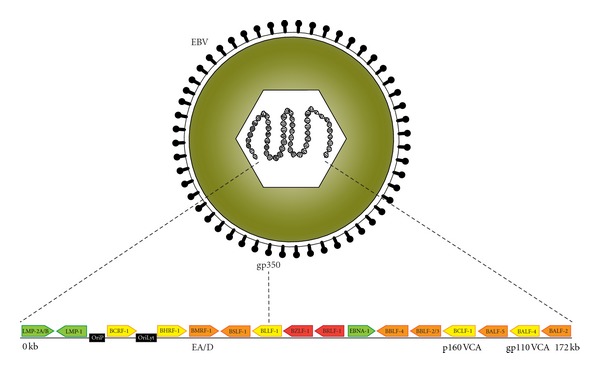
EBV structure and partial map of the genome. The EBV virion comprises a 172 kb linear dsDNA genome inside an icosahedral capsid enclosed by an envelope with viral glycoproteins (gp350) utilized for infection of B cells. The positions of the origins of latent and lytic replication of the viral genome, OriP and OriLyt, respectively, are illustrated in black boxes in the EBV genome map. Selected genes and their relative placement are shown as arrows pointed in the direction of translation [20]. In the latent state, only a few antigens are expressed including EBNA-1, LMP-1, and -2A/B (shown in green). Lytic replication begins with induction of the two early transcription factors (shown in red), which activate early viral promoters generating the initiation complex at OriLyt consisting of 6 viral gene products (illustrated in orange). During lytic cycle, various lytic antigens are expressed (shown in yellow) [20]. Gene products of *BMRF*-1, *BCLF*-1, and *BALF*-4 are depicted as EA/D, p160 VCA, and gp110 VCA, respectively.

EBV, also known as human herpesvirus 4 (HHV4), is comprised of a 172 kb linear dsDNA genome inside an envelope-enclosed icosahedral capsid (Figure 1). EBV is a ubiquitous infectious agent, latently infecting approximately 95% of the world's population [24]. It is transmitted via saliva and replicates initially at mucosal surfaces in oropharyngeal and nasopharyngeal epithelial cells, especially in the tonsillar area. Next, the virus enters the underlying tissues and infects resting B cells via binding of its viral envelope glycoprotein 350 (gp350) to the B-cell type 2 complement receptor (CD21) [25, 26].

Central to the understanding of EBV's disease biology is the ability of the virus to shift between an active lytic cycle and a latent state, from which the virus occasionally reactivates.

Primary EBV infection during childhood is asymptomatic, but infection in adolescence causes infectious mononucleosis (IM) in 30–70% of cases, where the virus infects up to 20% of the B cells in the body [27, 28]. The reason for the age-related difference in disease is unknown. The most common symptoms and clinical manifestations of IM are skin rash, palatal exanthema, arthralgias, renal disorders, anemia, granulocytopenia, thrombocytopenia, pharyngitis, lymphoadenopathy, hepatosplenomegaly, fatigue, muscle aches, fever, loss of appetite, headaches, and malaise. Furthermore, the central nervous system can be involved, including development of encephalitis or meningitis (Table 1) [29].

Many studies link EBV infection with various autoimmune diseases (e.g., SLE and multiple sclerosis [30–35]) and some cancers, including lymphoid malignancies (e.g., Burkitt's lymphoma [36]) and epithelial cell malignancies (e.g., nasopharyngeal carcinoma [37]). Several cutaneous manifestations have also been associated with EBV infection including hydroa vacciniforme, a photosensitivity dermatosis of childhood mediated by infiltrations of EBV-specific CD8^+^ cytotoxic T cells in the skin [38].

### 2.1. EBV Lytic Cycle

During the primary EBV infection, the virus is in its lytic cycle of existence. The early lytic genes, *BZLF-1* and *BRLF-1*, encoding two transcription factors, are essential for the induction of the lytic replication cycle of EBV and also in the reduction of promoter activity in the latent state of infection [39, 40]. They activate the early viral promoters required for generation of the initiation complex at the lytic origin of viral replication, *oriLyt*, consisting of 6 viral gene products. The 6 viral genes are *BALF-5*, encoding the viral DNA polymerase, *BMRF-1*, encoding Epstein-Barr virus diffuse early antigen (EBV-EA/D) [41–45], which is the viral DNA polymerase accessory protein, *BALF-2*, encoding a single-stranded DNA-binding protein, *BSLF-1* and *BBLF-4*, encoding the primase and the helicase, respectively, and *BBLF2/3*, which encodes the helicase-primase-associated protein [46–51]. The gathering of the initiation complex and the binding of the gene product of *BZLF-1* to *oriLyt *result in lytic replication of the virus.

During lytic cycle, the viral DNA is replicated by a mechanism, where the majority of the 90–100 viral genes are expressed [48]. Multiple rounds of replication are initiated within *oriLyt*, resulting in viral gene expression and viral genome replication with a 100- to 1000-fold amplification [26, 50]. This gives rise to the shedding of infectious virus into saliva that can infect other B cells and epithelial cells and also be transmitted to a new host [52].

Several lytic cycle antigens expressed during the lytic cycle of infection are involved in immune evasion. These include the *BCRF-1 *gene encoding a viral IL-10 homologue and *BHRF-1 *encoding restricted early antigen (EA/R), a viral Bcl-2 homologue. Like human IL-10, viral IL-10 inhibits the synthesis of interferon-*γ* (IFN-*γ*) and suppresses CD8^+^ cytotoxic T-cell responses and upregulation of MHC I expression [53]. EA/R protects both infected B cells and epithelial cells from apoptosis [54].

Another EBV lytic cycle antigen, EBV-EA/D, is localized both in the cytoplasm and in the nucleus of infected cells, where it colocalizes with the viral DNA polymerase. EBV-EA/D binds dsDNA without sequence specificity and is a part of the EBV DNA-binding complex together with the viral DNA polymerase. It is essential for the polymerase to replicate the viral genome, and EBV-EA/D is therefore termed the EBV DNA polymerase accessory protein [55–58]. EBV-EA/D is also demonstrated to be widely distributed on the newly synthesized EBV genome during lytic replication and is therefore suggested to stabilize the newly synthesized viral DNA [59]. 

In addition, EBV-EA/D has been shown to have transcription factor activity, inducing activation of several promoters downstream of the *oriLyt* component, which is required for viral lytic replication [45, 55]. Studies have proposed that EBV-EA/D somehow functions as a coactivator for the *BZLF-1 *gene product, improving its transactivation of both the *BALF-2* gene promoter [60] and the *BHLF-1 *gene promoter [44]. Different sites of the EBV-EA/D protein have been associated with its different functions. Amino acids 378–404 are required for its transactivator functions [61], and amino acids 194–238 are necessary for stimulation of the viral DNA polymerase [62].

Later in the lytic cycle of infection, the late lytic viral proteins are synthesized: EBV viral capsid antigen (EBV-VCA) and membrane antigen (MA). EBV-VCA is a protein composed of a 110 kDa glycoprotein (gp110) and a 160 kDa protein (p160) encoded by *BALF-4* and *BCLF-1*, respectively. gp110 is involved in virus maturation and improves the efficiency of the virus to infect B cells and epithelial cells [63], whereas p160 is essential for the assembly of the viral capsid [64].

### 2.2. Latent State

After primary infection, EBV usually enters the latent state as a consequence of the host's immune response. The result of primary EBV infection is numerous EBV-infected B cells, which have induced continuous proliferation and prevented apoptosis resulting in differentiation into immortalized resting memory B cells. These can exit the tonsils and enter the peripheral circulation, and they can persist for life in the host [52]. The EBV genomic DNA will undergo circularization and thus consists of a closed circular plasmid that behaves as the host's chromosomal DNA, which results in severely restricted expression of viral genes. Based on these and other immune evasion mechanisms, the virus becomes undetectable by the immune system [25, 26].

In the latent state of infection, nearly all of the approximately 80 viral promoters are silenced, and a maximum of 9 genes are expressed. These include the nuclear antigens (EBNA-1, -2, -3A, -3B, and -3C), the leader protein (LP), and the latent membrane proteins (LMP-1, -2A, and -2B) [52, 66]. LMP-1 and LMP-2A both act as survival signals of the infected B cell. LMP-1 serves as the signal that normally comes from the CD40 signal transduction pathway initiated by CD4^+^ T-cell help, and LMP-2A provides the signal normally generated by antigen binding of the B-cell receptor. Thus, these two latent EBV antigens rescue the infected B cells from apoptosis [66, 67].

EBNA-2 is known to be the most important transcription factor and controls the expression of all other latent viral genes. It blocks lytic replication in the majority of EBV-infected cells, ensuring the presence of latently infected B cells and thereby obstructing EBV elimination by the immune response of the host [52]. EBNA-1 is the only viral antigen required for maintenance of the viral genome as it acts as a replication factor during latent infection, where the EBV genomic DNA only is replicated once every cell cycle [26, 52]. When resting memory B cells are latently infected for a longer period of time, EBV only expresses EBNA-1. The EBNA-1 protein contains a glycine and alanine repeat domain, which ensures that the protein is not degraded by the proteasome of the host. Therefore, no EBNA-1 peptides are presented at the surface of the infected B cells, and the virus is thus hidden from the immune system [25, 68].

### 2.3. Reactivation and Switch to the Lytic Cycle

Occasionally, EBV can reactivate and switch back to the lytic cycle. The triggers for EBV reactivation are unknown. However, differentiation of infected B cells into plasma cells might trigger the activation of the promoter for early lytic genes, which eventually will result in replication and switch to lytic cycle [25, 52, 69]. Yet, the signals and timing involved in this process are unknown and must be a dynamic correlation between the host's immune response towards EBV and the infection state. It is established that activation of the lytic program happens in latently infected memory B cells passing through the lymphoid tissue associated with the pharynx mucosa [26]. Because of this ability of the virus to reactivate, it serves as a constant antigenic challenge to the host.

### 2.4. Response from the Immune System to EBV

Both latent and lytic EBV antigens are potent immunogens, and a vigorous immune response is initiated during EBV infection. This response involves all parts of the immune system and will control, but not eliminate, the infection. The expansion of EBV-infected B cells during lytic cycle is especially controlled by CD8^+^ cytotoxic T cells, which kill infected B cells and also induce the latent state in remaining EBV-infected B cells [70]. Cell-mediated immunity is also crucial in preventing the latent infection from entering lytic replication [25]. IFN-*γ* is suggested to be an important mediator of the immune response against EBV, as the level of IFN-*γ* is highly increased in patients with IM [71]. The clinical symptoms do not disappear until the amounts of both infected B cells in lytic cycle and of activated T cells are reduced, which occurs after approximately 4 weeks for normal immunocompetent individuals [25]. The CD8^+^ cytotoxic T-cell response toward EBV accounts for the cutaneous symptoms associated with EBV infection (Table 1) [72].

A humoral immune response is also initiated during EBV infection, and EBV-infected individuals have distinct serologic profiles during the latent and acute phases. In early stages of the primary infection, antibodies toward EBV-VCA and EBV-EA/D are generated, whereas EBNA-1 antibodies develop later. EBV-VCA IgM antibodies are diagnostic for recent active infection [73]. Antibodies of the IgG isotype to EBV-VCA and EBNA-1 will persist throughout life [74]. EBV-EA/D-directed antibodies are known as a strong indication of lytic replication of the virus [74]. Serum IgA antibodies toward the *BZLF-1 *gene product and EBV-EA/D have been shown to be produced during active disease and are suggested to be stimulated by EBV replication in mucosal sites [75]. The antibodies produced against EBV antigens counteract the viral infection mainly by antibody-dependent cell-mediated cytotoxicity [72].

## 3. Association between EBV Infection and SLE

Many studies have revealed a connection between SLE and EBV infection. Essentially all adult SLE patients are infected with EBV (99.5%) [24]. However, the statistical significance of this finding is reduced by the large proportion of healthy adults infected as well (95%). In young people below the age of 20 years, the difference between SLE patients and healthy controls is more evident, as the prevalence of EBV infection in the control population is lower, with only 70% being infected, while essentially all pediatric SLE patients are infected with EBV (99.6%) [76, 77].

As demonstrated in Table 1, SLE and EBV-induced IM are known to have similar symptoms and clinical manifestations, indicating an association. Most interesting, presence of rheumafactor and autoantibodies against cellular components like DNA, histones, and ribonucleoproteins is found in IM patients as well as in SLE patients [78, 79]. EBV infection may somehow result in both diseases according to the genetic predisposition and the immune response against EBV in the individual.

SLE patients have been shown to have at least a 10-fold increased frequency of EBV-infected peripheral B cells compared to healthy controls. This increase is associated with increased disease activity in SLE patients and is independent of intake of immunosuppressive medication [31]. In addition, an abnormally high viral load in the peripheral blood mononuclear cells (PBMCs) has been demonstrated in SLE patients compared to healthy controls in several studies [33–35, 80]. Kang et al. found, by the use of real-time quantitative PCR, a 40-fold increase of EBV load when comparing SLE patients to healthy controls [33], and Moon et al. found at least a 15-fold increase of EBV load in SLE patients [34]. Furthermore, Lu et al. found a significantly elevated level of EBV DNA in serum from 42% of the examined SLE patients compared to only 3% of the healthy controls [80]. These findings suggest EBV active lytic cycle with profound viral replication in SLE patients. Thus, it may be indicated that reactivation of EBV is associated with the development of SLE.

Studies on normal immunocompetent carriers of EBV demonstrate that they usually show little or no mRNA expression by EBV. Gross et al. have demonstrated that SLE patients have abnormal expression of 4 viral mRNAs: *BZLF-1, LMP-1, LMP-2*, and *EBNA-1* in their PBMCs [31]. The measured expression levels of mRNAs were often higher than in individuals with IM indicating very active virus. *BZLF-1* is one of the early lytic genes, facilitating the initiation of the lytic replication of the virus, and expression of this mRNA in SLE patients clearly indicates reactivation of the virus. In addition, an abnormal latency state is indicated in the SLE patients by the increased expression of the three latent state mRNAs. The enhanced expression of *LMP *mRNAs might result in improved survival of EBV-infected B cells, as the encoded antigens serve as survival signals that normally comes from the CD40 signal pathway and by antigen binding to the B-cell receptor [66, 67].

Also, Poole et al. measured the levels of EBV mRNA in PBMCs from SLE patients and healthy controls infected with EBV [81]. They found a 3.2-fold increase in the *BLLF-1 *mRNA encoding gp350, which is essential for the binding and infection of new B cells. Also, the *BCRF-1, EBNA-1*, and *LMP-2* mRNAs were increased 1.7-fold in SLE patients compared to healthy controls. These results suggest that the EBV infection is active and harder to control in the SLE patients.

Serologic evidence of a connection between EBV infection and SLE development has been illustrated several times by examining the presence of antibodies to EBNA-1, EBV-VCA, and EBV-EA in sera from SLE patients. Studies on antibodies to EBNA-1 and EBV-VCA are contradictive. Most studies show no difference in the prevalence of IgG and IgM antibodies to either EBNA-1 or EBV-VCA between SLE patients and healthy controls [82–85]. However, studies on pediatric SLE patients and one study on adults show that only two-thirds of healthy controls compared to all SLE patients are seropositive for these antibodies [24, 76, 77]. Furthermore, an elevated amount of both EBNA-1 and EBV-VCA IgA antibodies has been detected in SLE patients [80, 83, 86, 87].

In addition, elevated titers of IgG antibodies to early lytic antigens including EBV-EA/D, EBV-EA/R, and the *BALF-2* gene product have been found in approximately half of SLE patients compared to only 8–17% of healthy controls by several research groups [82, 84, 85, 88, 89]. Most interesting, elevated levels of IgA antibodies to these antigens have also been found in SLE patients, characteristic of epithelial infection. Lau et al. demonstrated that 15% of SLE patients compared to none of the healthy controls were positive for EBV-EA IgA antibodies by immunofluorescence [90], and Draborg et al. found a positive rate of IgA EBV-EA/D antibodies of 58% regarding SLE patients, whereas none of the serum samples from healthy controls showed IgA antibody binding to EBV-EA/D [65]. Furthermore, when compiling the positivity for EBV-EA/D-reacting antibody isotypes (IgG, IgM, and IgA) for each individual, 65% of the SLE patients were positive for two or three isotypes. None of the healthy controls were positive for three isotypes, and only 10% were positive for two isotypes, whereas the majority (65%) had no antibodies against EBV-EA/D (Table 2) [65]. These results could not be explained by intake of immunosuppressive medication, indicating that the antibodies do not occur upon reactivation of EBV due to an iatrogenically suppressed immune system. Presumably, the results of high prevalence of IgA antibodies against EBV reflect the host's attempt to control reactivation or reinfection of EBV in epithelial cells. Additionally, the presence of multiple EBV-EA/D antibody isotypes indicates a more disseminated EBV infection in SLE patients than in healthy controls.

The constant attempts of the host's immune system to control the virus apparently lead to attack on cells expressing EBV-EA/D, resulting in killing of infected cells before assembly of mature EBV particles. This results in release of EBV-EA/D (presumably bound to dsDNA) and intracellular antigens (including those involved in EBV replication and protein synthesis). Since this occurs both in B cells and epithelial cells, the antibody response involves both IgG and IgA to EBV-EA/D and autoantigens (e.g., dsDNA and ribonucleoproteins), depending on the individuals infection distribution and immune system.

Actually, IgA deficiency has been shown to be a risk factor for development of SLE as it results in frequent infections, and approximately 6% of SLE patients have been shown to suffer from IgA deficiency [91, 92]. It could be speculated that defects in controlling EBV infection result in SLE development in different ways. Presumably, some individuals develop SLE due to lack of production of IgA antibodies to counteract an epithelial EBV reactivation. Other individuals are not able to control EBV infection as a result of other immune defects and therefore attempt to control EBV with production of IgA antibodies against EBV lytic cycle antigens (especially EBV-EA/D).

An additional mechanism by which EBV can contribute to development of SLE is molecular mimicry. EBNA-1 has been shown to cross-react with SLE-associated autoantigens resulting in cross-reactive antibodies followed by epitope spreading, which eventually can result in development of SLE [93–95].

The reduced EBV-specific T-cell reactivity observed in SLE patients is possibly a consequence of defective EBV-specific T cells, which indicate poor control of EBV infection. Actually, the defective control of the virus has been demonstrated to involve an impaired EBV-specific T-cell response in SLE patients [33, 96]. Studies conducted by Berner et al. on EBV-specific T cells in SLE patients showed a tendency of an increased frequency of CD8^+^ T cells toward a specific epitope of the lytic cycle BMLF1 protein. These results were obtained by analysis of PBMCs using MHC I tetramers. Using an ELISPOT assay, the EBV-reactive CD8^+^ T cells were found to be incapable of producing IFN-*γ* upon stimulation [96]. This indicates that the EBV-specific CD8^+^ T cells of SLE patients may have a defect in their ability to become activated upon stimulation and will thereby produce poor effector responses. In addition, Kang et al. found a tendency of SLE patients to have a decreased amount of EBV-specific CD8^+^ cytotoxic T cells producing IFN-*γ*, when samples of whole blood were stimulated with EBV [33]. Simultaneously, they showed a significantly increased frequency of EBV-specific CD4^+^ T cells producing IFN-*γ* in SLE patients when stimulated [33]. These results suggest that the impaired EBV-specific T-cell response in SLE patients comprises a defect in EBV-specific CD8^+^ T-cell cytotoxicity and a compensatory increased frequency of EBV-specific CD4^+^ T cells.

The above-mentioned associations between active and uncontrolled EBV infection and SLE indicate that EBV and possibly also other viruses have a pathogenic role in the development of SLE. Other viruses besides EBV have been suggested to be associated with SLE including cytomegalovirus [97], parvovirus B19 [98], hepatitis B [98], and human endogenous retroviruses [99]. Overall, infections are presumably involved in SLE induction, and SLE patients have an increased susceptibility to many kinds of infections [100]. These findings are related to the intrinsic immune defects found in SLE patients.

## 4. Conclusion

The much investigated association between EBV infection and the development of SLE indicates genetic and/or acquired difficulties with suppressing the infection and keeping EBV in its latent state. This is demonstrated by defective EBV-specific T cells, an abnormally high viral load, expression of viral genes, and high levels of EBV IgA antibodies in SLE patients.

Presently, the major genetic predisposing factors are deficiencies in components of the classical complement pathway [17], certain MHC alleles [5], components of IFN pathways, and other immune-regulatory pathways [9, 10, 91]. Acquired antibodies to C1q may also contribute to disease development. These factors may contribute in different ways. Genetically determined immune deficiencies and the presence of particular MHC alleles may first of all limit the ability to control EBV infection and reactivation, and complement deficiencies impair the removal of necrotic and apoptotic cell debris [14–16, 18]. This theory covers many aspects of SLE, but does not explain the female preponderance. Presumably, the solution to this problem shall be found in genetically determined immune system differences or in pregnancy/maternity-related influences on the immune system.

**Figure 2:**
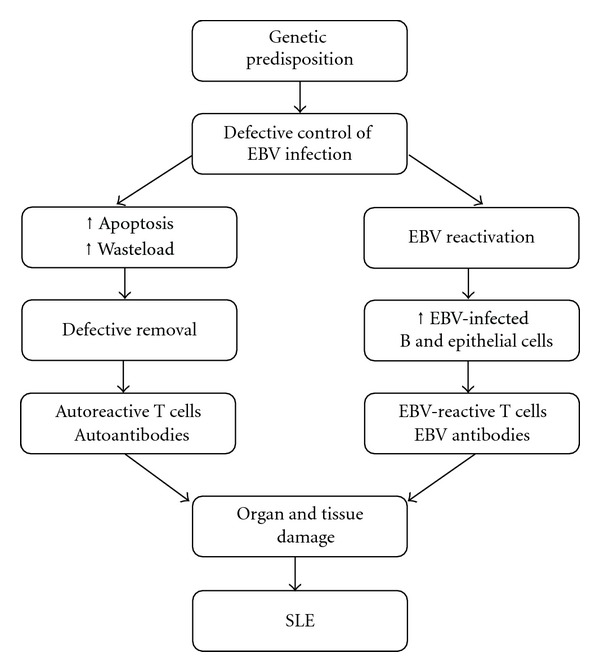
Hypothesis of development of SLE from EBV infection. Genetic insufficiencies may result in poor control and thereby more frequent reactivation of the latent EBV infection. The increased number of EBV-infected cells will, upon apoptosis, initiate an innate and adaptive immune response against the released cellular antigens and EBV antigens due to defective removal of waste products. This will result in production of autoantibodies and EBV antibodies as an attempt to control the virus-induced inflammation. Furthermore, activation of both autoreactive and EBV-reactive T cells will occur. The response from the immune system cause organ and tissue damage leading to development of SLE.

As demonstrated in Figure 2, it is hypothesized that lack of control of EBV infection could result in more widespread latent infection and more frequent reactivation. This entails increased numbers of EBV-infected B cells and epithelial cells and may lead to enhanced apoptosis of cells and amplified cellular waste load. An immune response is therefore initiated with development of autoantibodies against cell components. Lack of control of EBV infection may thus be a contributing factor to development of SLE. EBV reactivation may also give rise to release of EBV lytic cycle antigens resulting in the demonstrated production of EBV-directed antibodies reflecting the hosts attempt to control the reactivation.

Clinically, the constant interplay between EBV reactivation, reinfection, and the host's immune response results in individual disease patterns and clinical presentations, spanning from initial mild symptoms to ultimate classification as SLE as more and more ACR criteria are fulfilled.

In conclusion, the demonstrated associations between EBV and SLE suggest that infection with and reactivation of EBV has a pathogenic role as an environmental trigger inducing or promoting the development of SLE in genetically predisposed individuals.

## Figures and Tables

**Table 1:** Symptoms and clinical manifestations of SLE* [3, 4, 6] and IM [29].

SLE	IM
**Malar rash**	Skin rash
**Discoid rash**	Palatal exanthema
**Photosensitivity**	
**Oral/nasopharyngeal ulcers**	Pharyngitis
**Arthritis**	Arthralgias
**Serositis**	
**Renal disorders**	Renal disorders
**Hematologic disorders**	
** Anemia**	Anemia
** Leucopenia**	Granulocytopenia
** Lymphocytopenia**	
** Thrombocytopenia**	Thrombocytopenia
**Immunological disorders**	Lymphoadenopathy
**ANAs**	ANAs
** Anti-dsDNA**	Anti-DNA
** Anti-Sm **	
Anti-histone	Anti-histone
Anti-ribonucleoprotein	Anti-ribonucleoprotein
Rheumafactor	Rheumafactor
**Neurologic disorders (seizures/psychosis)**	Neurological disorders (encephalitis/meningitis)
	Headaches
Fatigue	Fatigue
Muscle aches	Muscle aches
Low-grade fever	Fever
Loss of appetite	Loss of appetite
	Malaise
	Hepatosplenomegaly

*ACR criteria highlighted in bold.

**Table 2:** EBV-EA/D antibodies in SLE patients and healthy controls [65].

No. of antibody isotypes (IgG, IgA, IgM)	% SLE patients	% Healthy controls
0	12%	65%
1	23%	25%
2	28%	10%
3	37%	0%

## Abbreviations

ACR: American College of Rheumatology
ANA: Antinuclear antibody
dsDNA: Double-stranded DNA
EA/D: Diffuse early antigen
EA/R: Restricted early antigen
EBNA: Epstein-Barr nuclear antigen
EBV: Epstein-Barr virus
gp: Glycoprotein
HHV4: Human herpesvirus 4
IFN: Interferon
IL: Interleukin
IM: Infectious mononucleosis
LMP: Latent membrane protein
LP: Leader protein
MA: Membrane antigen
MHC: Major histocompatibility complex
PBMC: Peripheral blood mononuclear cell
SLE: Systemic lupus erythematosus
VCA: Viral capsid antigen.
